# Body Shape Index Is a Stronger Predictor of Diabetes

**DOI:** 10.3390/nu11051018

**Published:** 2019-05-07

**Authors:** Hiba Bawadi, Merna Abouwatfa, Sara Alsaeed, Abdelhamid Kerkadi, Zumin Shi

**Affiliations:** Department of Nutrition, College of Health Sciences, QU-Health, Qatar University, Doha P.O. Box 2713, Qatar; a1409072@student.qu.edu.qa (M.A.); ma1403220@student.qu.edu.qa (S.A.); abdel.hamid@qu.esdu.qa (A.K.); zumin@qu.edu.qa (Z.S.)

**Keywords:** ABSI, BMI, Qatar Biobank, Diabetes

## Abstract

Anthropometric indicators can predict the development of diabetes among adults. Among them, a new indicator (Body Shape Index) was developed. Several cohort observational studies have demonstrated that A Body Shape Index (ABSI) is a prominent indicator for mortality and morbidity. Nevertheless, the predictive level of ABSI for diabetes varied among different ethnicities. This study aimed to assess the predictive level of ABSI for diabetes compared to BMI in the Qatari population. Date from 2536 Qatari adults aged 20–79 years attending the Qatar Biobank Study were used. Body height, weight, and waist circumference were measured. Blood samples were measured for glucose. The association between ABSI, BMI, and diabetes was assessed using a logistic regression. Both ABSI and BMI were positively associated with diabetes after adjusting for potential confounding factors. ABSI had a stronger association with diabetes than BMI. Per 1 SD increment of ABSI and BMI, the z-score had an odds ratios of 1.85 (1.54–2.23) and 1.34 (1.18–1.51) for diabetes, respectively. ABSI and BMI are significantly associated with diabetes in the Qatari population. ABSI is a better predictor for the risk of diabetes than BMI after the adjustment for age, gender, education, and physical activity.

## 1. Introduction

The prevalence of diabetes mellitus is high among Qatari adults reaching 16.7% [1]. The progression of type 2 diabetes is accompanied with several complications such as neuropathy, retinopathy, cardiovascular disease, infections (e.g., monilial skin infections), and cognitive impairment [2]. The increase in the Type 2 diabetes prevalence is associated with a change in sedentary life style and obesity [3]. Different researches demonstrated that obesity, particularly abdominal obesity (AO), which represents both subcutaneous and visceral fat accumulation, is associated with an increased risk of cardiovascular and metabolic diseases in both adults and children [4]. It is well-known that obesity is linked with insulin resistance, which plays an important role in the pathophysiology of type 2 diabetes and other metabolic disorders [5,6]. Body mass index (BMI) is a widely accepted and easily applicable measure for obesity; it, however, cannot distinguish between fat and fat-free mass. Therefore, an elevated BMI might not necessarily reflect an increased adiposity [7]. To overcome this weakness, waist circumference (WC) has been used as indicators of abdominal obesity and is closely related to noncommunicable diseases [8,9]. It has been shown that an excess of WC is associated with an increase in the prevalence of cardiovascular diseases (CVD) and the risk of premature death even when BMI is within a normal range [10]. It was also reported that the association between insulin resistance and WC is better than that with BMI [11,12]. In 2012, Krakauer and Krakauer developed a new tool called a body shape index (ABSI) that accompanies waist circumference, height, and weight [13]. According to the authors, ABSI emphasizes the elevated risk of diseases related to central and general adiposity. Therefore, ABSI is capable of depicting a crucial risk factor for premature mortality [13]. ABSI showed a predictive power to the risk of CVD and cancer [14]. ABSI can estimate both visceral abdominal and general overall adiposities and can predict premature mortality better than WC and BMI [15]. ABSI has been validated to predict the risk of diabetes in several countries. However, the findings are mixed. Some studies suggest that ABSI is a better predictor for diabetes [16,17]. However, other studies showed ABSI is not a better predictor of diabetes than BMI [18].

The aim of this study is to assess the predictive level of ABSI for diabetes compared to BMI in the Qatari population. We also aimed to assess the interaction between ABSI and BMI in relation to diabetes.

## 2. Methodology

### 2.1. Study Design

The study is a population-based cross-sectional survey carried out between 2012–2014 for 60,000 men and women Qatari nationals and long-term residents (individuals living in the country for ≥15 years) (Qatar Biobank study); the detailed methods of data collection and sampling has been published elsewhere [19]. Exclusion criteria were a history of terminating diseases such as muscle wasting, athletes, age <20 or >79, and pregnant women. A random sample of 2802 adults aged 20–79 years was obtained from the Qatar Biobank survey data. Participants with incomplete measurements were excluded (*n* = 266). The final number of participants included in this analysis was 2536 (1275 males and 1261 females). Qatar Biobank recruitment and data collection protocols were approved by the Hamad Medical Corporation Ethics Committee. The Institutional Review Board approval for this study was obtained from Qatar Biobank.

### 2.2. Outcome Variable Diabetes

Diabetes was defined as having fasting glucose ≥7 mmol/dL, random glucose ≥11.1 mmol/dL, HbA1c >6.5%, or self-reported doctor diagnosed diabetes [20]. Anthropometric measurements including weight, height, and waist circumference were measured by competent registered nurses in Qatar Biobank clinics. The participants were asked to wear light clothing and to be barefoot. The weight was measured by kg and recorded to the closest 0.1 kg. The standing and sitting height were measured and recorded to the closest 0.1 cm with the subject’s head in the Frankfurt plane. The waist circumference (WC) was measured at the midpoint between the last rib and the top of the iliac crest with stretch-resistant tape; the participants stood with their feet close together, arms positioned on the side. The weight and height measures were used to calculate body mass index (BMI, kg/m^2^). The BMI status (normal, overweight, and obese) of the participants were assigned based on WHO BMI cutoff points [21]. DEXA-Full Body iDXA (GE) scan-scanners were used in measuring the body distribution of visceral and trunk fat. It works by passing a low dose of x-ray radiation that goes on the whole body of participants who were directed not to wear anything that might interfere with the x-ray of the device like metal belts or jewelry. They were asked to wear a gown and to lie flat on the screening for about 5 to 10 min, and for clear not blurry Full Body iDXA (GE) scan images, participants were asked to hold still without moving and to hold their breath for a few seconds.

A body shape index (ABSI) was calculated using the Krakauer and Krakauer equation: WC/(BMI^2/3^height^1/2^) [13]. The ABSI score was converted to a z-score using the following equation: ABSI minus ABSI_mean_ divided by ABSI_SD._ BMI was also converted to z-score. The z score was used to measure the association of ABSI compared with BMI and the risk of diabetes. The z-score was used as it makes the comparison meaningful because the unit change in the regression analysis is different for ABSI and BMI.

### 2.3. Covariates

The education level, age, and physical activity of the participants were obtained through a main questionnaire. Education was divided into three levels: Low education (up to secondary school), medium education (technical or professional school), and high education (university and above). Leisure time physical activity levels (expressed as metabolic equivalents (MET) (hours/week) were calculated based on the frequency and duration of different types of physical activity.

### 2.4. Statistical Analysis

The association between ABSI z-scores and diabetes was assessed using a logistic regression. Four models were used: model 1 adjusted for age and gender; model 2 further adjusted for education; model 3 further adjusted for physical activity; model 4 further adjusted for BMI. The association between BMI (z-score) and diabetes was also assessed using the same approach. Subgroup analyses were conducted and visually presented using a user-written syntax ipdover in Stata. All the analyses were conducted in Stata 15.1. A *p* value < 0.05 was considered as statistically significant.

## 3. Results

Table 1 shows the sample characteristics by gender. The mean age was 38.3 in males and 40.1 in females. More than 60% of the participants had a high education level. The prevalence of obesity was 39% (32.6% in men and 45.4% in women). Females had higher mean BMI and total fat mean than males. Males had higher waist circumference and visceral fat (*p* < 0.001). Sample characteristics by incidence of diabetes is presented Table S1.

Table 2 shows the association between ABSI and BMI with diabetes using different logistic regression models with progressive adjustments. After adjusting for age and gender, both ABSI and BMI were positively associated with diabetes with an OR (95% CI) of 1.84 (1.54–2.21) and 1.38 (1.22–1.56) respectively. With a further adjustment for education, the OR for the risk of diabetes with ABSI z-scores was 1.87 (95% CI: 1.55–2.24), whereas the OR for the association between the risk of diabetes and BMI z-scores was 1.34 (95% CI: 1.55–1.19–1.52). After being mutually adjusted for ABSI and BMI, the ORs for diabetes increased for both ABSI 1.94 (95% CI: 1.61–2.34) and BMI 1.40 (95% CI: 1.24–1.60). The association between ABSI, BMI and HbA1c was assessed using a multivariable regression model where both indices were independently associated with HbA1c (refer to Figure S1 in the Supplementary Materials).

**Figure 1:**
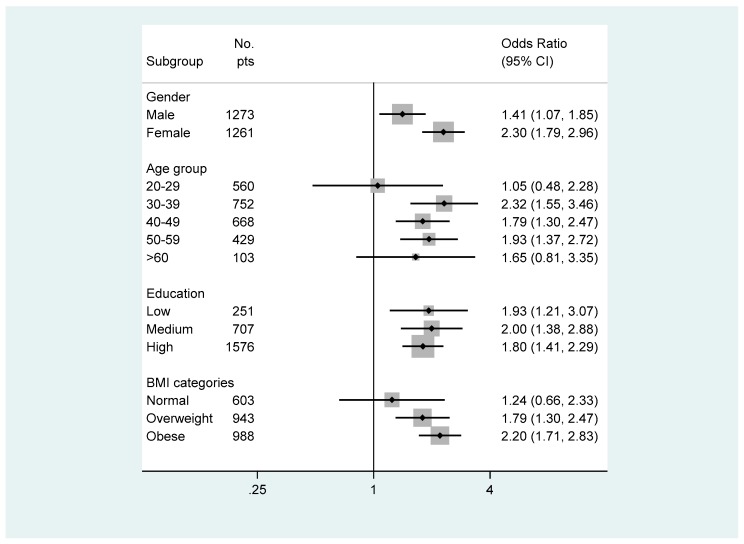
Subgroup analyses of the association between ABSI and diabetes.

Figure 1 represents subgroup analyses of the association between ABSI and diabetes. There was a significant interaction between gender and ABSI in relation to diabetes. The association between ABSI and diabetes was stronger in women than in men. ABSI and diabetes were associated in all subgroups of age ≥30. The highest association were observed in the second age (30–39 years) subgroup (*p* < 0.001). Participants with a normal weight did not show a significant association between ABSI and diabetes while overweight and obese did (*p* < 0.001); however, obese participants showed the highest association between ABSI and diabetes (*p* < 0.001). As BMI became higher, the OR gradient for diabetes associated with ABSI also became higher. The OR for diabetes associated with ABSI were 1.24, 1.79, and 2.20 among normal, overweight, and obese participants.

## 4. Discussion

This cross-sectional study assessed the predictive power of ABSI and BMI on predicting the risk of diabetes in the Qatari population. It was found in the current study that ABSI is associated with a risk of diabetes to a greater extent as compared to BMI. Han et al. (2017) compared the predictive level of a body shape index (ABSI) to other anthropometric parameters including body mass index (BMI), waist to height ratio (WHtR), and waist circumference (WC) for the risk of developing type 2 diabetes mellitus (T2DM) in chinses adults [22]. After adjusting for different cofounding variables, ABSI differentiated between the cases and non-cases of diabetes; however, there was no difference in the predictive ability for T2DM when using ABSI or other anthropometric parameters [22]. Fujita and colleagues (2015) conducted a prospective cohort study aimed at addressing whether ABSI could be used as a predictor for the risk of developing type 2 diabetes mellitus and other chronic disease [23]. Researchers used annual health examination data (2008 to 2012) from Chiba City Hall in Japan. Their study included 37581 nondiabetic subjects followed up for 4 years. An increased BMI, WC, and ABSI elevated the risk of T2DM. Furthermore, the areas under the curve associated with ABSI regression models were smaller than that for BMI or WC models, which indicates that ABSI may not a stronger predictor for diabetes among Japanese population [23]. Similar findings were reported from an analysis of data from an 11-year follow-up study [24]. The authors reported that ABSI was an inferior discriminator of incident T2DM as compared to BMI and other anthropometric measures.

Interestingly, we found that the joint effect of BMI and ABSI is a better predicator for the risk of diabetes than the ABSI and BMI alone. This finding is in line with a recent retrospective study about the association between Body Shape Index (ABSI) with cardio-metabolic risk factors [16]. The study was conducted on 6081 Caucasian adults. Generalized linear models (GLM) were used to assess both sex and age and the adjusted association of the ABSI with binary and continuous cardio-metabolic risk factors. The results showed that the joint contribution of BMI and ABSI produced significantly improved associations for having a high fasting glucose, high triglycerides, and a low HDL but not high blood pressure [16].

## 5. Conclusions

ABSI and BMI are significantly associated with diabetes in the Qatari population. ABSI is a better predictor for the risk of diabetes than BMI after the adjustment for age, gender, education, and physical activity.

## Figures and Tables

**Table 1:** Sample characteristics by gender.

	Total	Male	Female	*p*-Value
	*n* = 2536	*n* = 1275	*n* = 1261	
Age (years)	39.4 (11.1)	38.8 (10.6)	40.1 (11.6)	0.004
Education				<0.001
Low	251 (9.9%)	94 (7.4%)	157 (12.5%)	
Medium	707 (27.9%)	388 (30.5%)	319 (25.3%)	
High	1576 (62.2%)	791 (62.1%)	785 (62.3%)	
BMI (kg/m^2^)	29.0 (5.6)	28.4 (5.0)	29.6 (6.1)	<0.001
BMI categories				<0.001
Normal	603 (23.8%)	316 (24.8%)	287 (22.8%)	
Overweight	944 (37.2%)	543 (42.6%)	401 (31.8%)	
Obese	989 (39.0%)	416 (32.6%)	573 (45.4%)	
Waist circumference (cm)	89.2 (13.5)	93.5 (12.4)	84.8 (13.2)	<0.001
ABSI z score	0.0 (1.0)	0.7 (0.7)	−0.7 (0.7)	<0.001
Total mass (kg)	79.3 (16.3)	84.3 (15.2)	73.5 (15.7)	<0.001
Total fat (kg)	30.9 (10.7)	28.6 (10.3)	33.4 (10.6)	<0.001
Trunk fat (kg)	16.2 (6.4)	16.2 (6.6)	16.2 (6.2)	0.90
Visceral fat (kg)	1.0 (0.7)	1.3 (0.8)	0.8 (0.5)	<0.001
Leisure time physical activity (MET hours/week)	18.2 (38.6)	22.4 (45.3)	13.9 (29.8)	<0.001
Diabetes (%)	413 (16.3%)	191 (15.0%)	222 (17.6%)	0.073

The data are presented as mean (SD) for continuous measures and *n* (%) for categorical measures.

**Table 2:** The association between A Body Shape Index (ABSI) and BMI with diabetes among Qatari adults.

	ABSI z-Score		BMI z-Score	
	OR (95% CI)	*p*	OR (95% CI)	*p*
Model 1	1.84 (1.54–2.21)	<0.001	1.38 (1.22–1.56)	<0.001
Model 2	1.87 (1.55–2.24)	<0.001	1.34 (1.19–1.52)	<0.001
Model 3	1.85 (1.54–2.23)	<0.001	1.34 (1.18–1.51)	<0.001
Model 4	1.94 (1.61–2.34)	<0.001	1.40 (1.24–1.60)	<0.001

Model 1 adjusted for age and gender; Model 2 further adjusted for education; Model 3 further adjusted for physical activity; Model 4 further adjusted for the BMI z-score or ABSI z-score.

## Supplementary Materials

The following are available online at https://www.mdpi.com/2072-6643/11/5/1018/s1, Figure S1: Association between ABSI, BMI and HbA1c; Model adjusted for age and gender. ABSI and BMI were mutually adjusted. Table S1: Sample characteristics by diabetes.

## References

[1] Shaw J.E., Sicree R.A., Zimmet P.Z. (2010). Global estimates of the prevalence of diabetes for 2010 and 2030. Diabetes Res. Clin. Pract..

[2] Massi-Benedetti M. (2002). The cost of diabetes in Europe-Type II: The CODE-2 study. Diabetologia.

[3] Tuomilehto J., Lindström J., Eriksson J.G., Valle T.T., Hämäläinen H., Ilanne-Parikka P., Keinänen-Kiukaanniemi S., Laakso M., Louheranta A., Rastas M. (2001). Prevention of type 2 diabetes mellitus by changes in lifestyle among subjects with impaired glucose tolerance. N. Engl. J. Med..

[4] Britton K.A., Massaro J.M., Murabito J.M., Kreger B.E., Hoffmann U., Fox C.S. (2013). Body fat distribution, incident cardiovascular disease, cancer, and all-cause mortality. J. Am. Coll. Cardiol..

[5] Orgel E., Mittelman S.D. (2013). The links between insulin resistance, diabetes, and cancer. Curr. Diab. Rep..

[6] Ye J. (2013). Mechanisms of insulin resistance in obesity. Front. Med..

[7] Kok P., Seidell J., Meinders A. (2004). The value and limitations of the body mass index (BMI) in the assessment of the health risks of overweight and obesity. Ned. Tijdschr. Geneeskd..

[8] Feller S., Boeing H., Pischon T. (2010). Body mass index, waist circumference, and the risk of type 2 diabetes mellitus: Implications for routine clinical practice. Dtsch. Arztebl. Int..

[9] Flint A.J., Rexrode K.M., Hu F.B., Glynn R.J., Caspard H., Manson J.E., Willett W.C., Rimm E.B. (2010). Body mass index, waist circumference, and risk of coronary heart disease: A prospective study among men and women. Obes. Res. Clin. Pract..

[10] Cerhan J.R., Moore S.C., Jacobs E.J., Kitahara C.M., Rosenberg P.S., Adami H.-O., Ebbert J.O., English D.R., Gapstur S.M., Giles G.G. (2014). A pooled analysis of waist circumference and mortality in 650,000 adults. Elsevier.

[11] Balkau B., Deanfield J.E., Després J.-P., Bassand J.-P., Fox K.A., Smith S.C., Barter P., Tan C.-E., Van Gaal L., Wittchen H.-U. (2007). CLINICAL PERSPECTIVE. Circulation.

[12] Sumner A.E., Sen S., Ricks M., Frempong B.A., Sebring N.G., Kushner H. (2008). Determining the waist circumference in African Americans which best predicts insulin resistance. Obesity.

[13] Krakauer N.Y., Krakauer J.C. (2012). A new body shape index predicts mortality hazard independently of body mass index. PLoS ONE.

[14] Song X., Jousilahti P., Stehouwer C., Söderberg S., Onat A., Laatikainen T., Yudkin J., Dankner R., Morris R., Tuomilehto J. (2015). Cardiovascular and all-cause mortality in relation to various anthropometric measures of obesity in Europeans. Nutr. Metab. Cardiovasc. Dis..

[15] Lee D.Y., Lee M.Y., Sung K.C. (2018). Prediction of mortality with a body shape index in young Asians: Comparison with body mass index and waist circumference. Obesity.

[16] Bertoli S., Leone A., Krakauer N.Y., Bedogni G., Vanzulli A., Redaelli V.I., De Amicis R., Vignati L., Krakauer J.C., Battezzati A. (2017). Association of Body Shape Index (ABSI) with cardio-metabolic risk factors: A cross-sectional study of 6081 Caucasian adults. PLoS ONE.

[17] He S., Chen X. (2013). Could the new body shape index predict the new onset of diabetes mellitus in the Chinese population?. PLoS ONE.

[18] Wu Y., Ding Y., Tanaka Y., Zhang W. (2014). Risk factors contributing to type 2 diabetes and recent advances in the treatment and prevention. Int. J. Med. Sci..

[19] Al Kuwari H., Al Thani A., Al Marri A., Al Kaabi A., Abderrahim H., Afifi N., Qafoud F., Chan Q., Tzoulaki I., Downey P. (2015). The Qatar Biobank: Background and methods. BMC Public Health.

[20] Association A.D. (2018). 2. Classification and diagnosis of diabetes: Standards of medical care in diabetes—2018. Diabetes Care.

[21] World Health Organization (2008). Waist Circumference and Waist–Hip Ratio: Report of a WHO Expert Consultation.

[22] Han C., Liu Y., Sun X., Luo X., Zhang L., Wang B., Ren Y., Zhou J., Zhao Y., Zhang D. (2017). Prediction of a new body shape index and body adiposity estimator for development of type 2 diabetes mellitus: The Rural Chinese Cohort Study. Br. J. Nutr..

[23] Fujita M., Sato Y., Nagashima K., Takahashi S., Hata A. (2015). Predictive power of a body shape index for development of diabetes, hypertension, and dyslipidemia in Japanese adults: A retrospective cohort study. PLoS ONE.

[24] Hardy D.S., Stallings D.T., Garvin J.T., Xu H., Racette S.B. (2017). Best anthropometric discriminators of incident type 2 diabetes among white and black adults: A longitudinal ARIC study. PLoS ONE.

