# Supplementary material for: Body Shape Index Is a Stronger Predictor of Diabetes
Source: Nutrients. 2019 May 7;11(5):1018. doi: 10.3390/nu11051018 (PMC6566958; doi:10.3390/nu11051018)
Supplement: Supplementary file 1 [file nutrients-11-01018-s001.pdf]

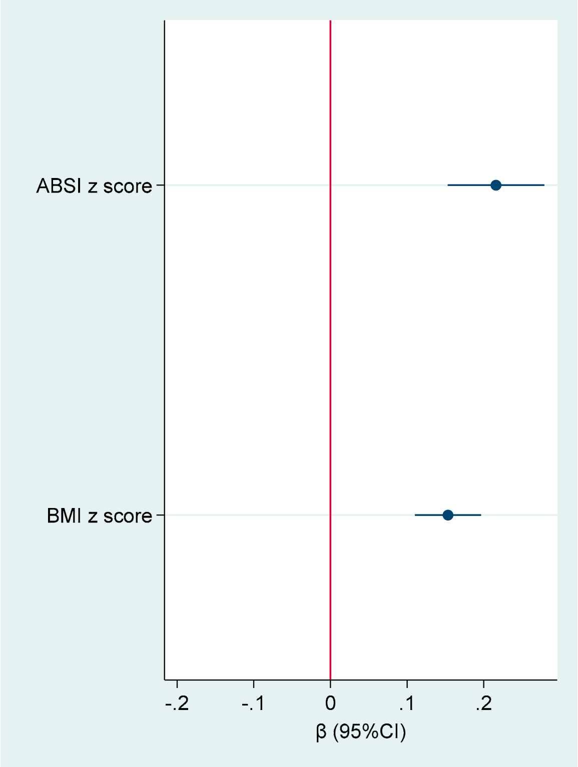

Figure S1. Association between ABSI, BMI and HbA1c; Model adjusted for age and gender. ABSI and BMI were mutually adjusted.

Table S1. Sample characteristics by diabetes.

	Total N=2,536	No N=2,123	Yes N=413	p-value
Sex				0.073
Male	1,275 (50.3%)	1,084 (51.1%)	191 (46.2%)	
Female	1,261 (49.7%)	1,039 (48.9%)	222 (53.8%)	
Age (years)	39.4 (11.1)	37.5 (10.3)	49.2 (9.8)	<0.001
RECODE of education				<0.001
Low	251 (9.9%)	143 (6.7%)	108 (26.2%)	
Medium	707 (27.9%)	609 (28.7%)	98 (23.7%)	
High	1,576 (62.2%)	1,369 (64.5%)	207 (50.1%)	
BMI (kg/m2)	29.0 (5.6)	28.5 (5.5)	31.4 (5.6)	<0.001
RECODE of bmi				<0.001
Normal	603 (23.8%)	564 (26.6%)	39 (9.4%)	
Overweight	944 (37.2%)	797 (37.5%)	147 (35.6%)	
Obese	989 (39.0%)	762 (35.9%)	227 (55.0%)	
Waist circumference (cm)	89.2 (13.5)	87.6 (13.1)	97.3 (12.5)	<0.001
ABSI z score	0.0 (1.0)	-0.0 (1.0)	0.3 (1.0)	<0.001
Total mass (kg)	79.3 (16.3)	78.5 (16.2)	83.3 (16.3)	<0.001
Total fat (kg)	30.9 (10.7)	30.2 (10.5)	34.1 (11.1)	<0.001
Trunk fat (kg)	16.2 (6.4)	15.6 (6.2)	19.1 (6.7)	<0.001
Total visceral fat (kg)	1.0 (0.7)	0.9 (0.7)	1.5 (0.8)	<0.001
Leisure time physical activity (MET hours/week)	18.2 (38.6)	19.2 (40.2)	13.0 (28.7)	0.003
